# Revealing the Therapeutic Potential of Botulinum Neurotoxin Type A in Counteracting Paralysis and Neuropathic Pain in Spinally Injured Mice

**DOI:** 10.3390/toxins12080491

**Published:** 2020-07-31

**Authors:** Valentina Vacca, Luca Madaro, Federica De Angelis, Daisy Proietti, Stefano Cobianchi, Tiziana Orsini, Pier Lorenzo Puri, Siro Luvisetto, Flaminia Pavone, Sara Marinelli

**Affiliations:** 1 CNR—National Research Council, Institute of Biochemistry and Cell Biology, 00015 Monterotondo Scalo (RM), Italy; valentina.vacca@ibcn.cnr.it (V.V.); tiziana.orsini@cnr.it (T.O.); siro.luvisetto@cnr.it (S.L.); 2 IRCCS Santa Lucia Foundation, 00143 Roma, Italy; luca.madaro@uniroma1.it (L.M.); fede75deangelis@gmail.com (F.D.A.); daisy.proietti@gmail.com (D.P.); 3 DAHFMO-Unit of Histology and Medical Embryology, Sapienza University of Rome, 00161 Rome, Italy; 4 Institute of Neurosciences and Department of Cell Biology, Physiology and Immunology, Universitat Autonoma de Barcelona, E-08193 Bellaterra, Spain; cobianchi.stefano@gmail.com; 5 Development, Aging and Regeneration Program, Sanford Burnham Prebys Medical Discovery Institute, La Jolla, CA 92037, USA; lpuri@sbpdiscovery.org

**Keywords:** botulinum neurotoxin type A, glial scar, spinal cord injury, neuropathic pain, neurodegeneration, paralysis, regeneration

## Abstract

Botulinum neurotoxin type A (BoNT/A) is a major therapeutic agent that has been proven to be a successful treatment for different neurological disorders, with emerging novel therapeutic indications each year. BoNT/A exerts its action by blocking SNARE complex formation and vesicle release through the specific cleavage of SNAP-25 protein; the toxin is able to block the release of pro-inflammatory molecules for months after its administration. Here we demonstrate the extraordinary capacity of BoNT/A to neutralize the complete paralysis and pain insensitivity induced in a murine model of severe spinal cord injury (SCI). We show that the toxin, spinally administered within one hour from spinal trauma, exerts a long-lasting proteolytic action, up to 60 days after its administration, and induces a complete recovery of muscle and motor function. BoNT/A modulates SCI-induced neuroglia hyperreactivity, facilitating axonal restoration, and preventing secondary cells death and damage. Moreover, we demonstrate that BoNT/A affects SCI-induced neuropathic pain after moderate spinal contusion, confirming its anti-nociceptive action in this kind of pain, as well. Our results provide the intriguing and real possibility to identify in BoNT/A a therapeutic tool in counteracting SCI-induced detrimental effects. Because of the well-documented BoNT/A pharmacology, safety, and toxicity, these findings strongly encourage clinical translation.

## 1. Introduction

Traumatic spinal cord injury (SCI) represents a dramatic health and social challenge that needs urgent attention by the medical and scientific community. SCI causes loss of sensorimotor functions and the associated comorbidities, including neuropathic pain (NeP) [1]. A global incidence of 10.5 every 100,000 people/year was estimated [2,3]. These data give an idea of the vastness of the problem with a huge economic and social impact (9.7 billion dollars—Centers for Disease Control and Prevention, USA). The main causes of SCI are road, domestic, sports, weapons, and at-work accidents.

Pathophysiology of SCI is characterized by primary and secondary phases. The first is related to the mechanical impact, which induces local neurons and glia death within minutes to hours. Acute, intermediate, and chronic stages characterize the secondary phase. In the acute phase, starting a few minutes after trauma, pathophysiological changes (edema, thrombosis, inflammation, and neuronal excitotoxicity) occur, giving rise to neuroinflammatory response. In the intermediate stage, from days to weeks post-injury, cell degenerative mechanisms, which include demyelination, microglial, and macrophage activation and apoptosis, gradually spread out from the lesion epicenter to uninvolved neural areas, causing further nervous tissue loss. Phenomena that should limit the damage and facilitate recovery process create a loop: on the one hand, the clearance of tissue debris (microglia activation and immune cell infiltration) and curbing the cell death area (glial scar formation) appear crucial to counteract the damage; on the other hand, the excess of proinflammatory agents, released from activated microglia and hyperactive astrocytes, as well as the uncontrolled oxidative stress, ischemia, and glutamate-mediated excitotoxicity, cause cell death. Moreover, the glial scar formation surrounding the necrotic part of epicenter represents a physical barrier that hampers the neural function and axonal regeneration. Finally, chronic phase is characterized by maturation of the lesion with glia scar and syrinx development [4].

The induction of neuroplasticity after SCI in adult CNS is considered a challenge; to date, no therapy is able to reverse the consequences of SCI [5]. Nonetheless, both preclinical and clinical evidence demonstrated that a certain degree of neurological recovery is possible [6].

Considering the complexity of the molecular and cellular environment of SCI, an ideal drug should be able to simultaneously target different components to permit spontaneous neural circuits’ restoration. Under these premises, we identified in botulinum neurotoxin serotype A (BoNT/A), for its intrinsic properties, a new therapeutic tool [7] for counteracting SCI. We tested toxin efficacy for a long time (30/60 days) in (i) a new validate severe SCI mouse model, which induces a complete long-lasting paralysis [8]; and (ii) a classical model of moderate SCI, which induces a short-term motor dysfunction and does not affect retraction and pain sensitivity, thus permitting to investigate NeP.

The BoNT/A therapeutic utilization has successfully expanded, becoming the most widely used biological drug [9]. Botulinum neurotoxins (BoNTs) act by inhibiting vesicle fusion and neurotransmission. Each neurotoxin’s serotype (A–G) cleaves to one of the SNARE-complex proteins, avoiding exocytosis (BoNT/A cuts SNAP25).

BoNT/A affects PNS and CNS. It is able to be retrogradely transported in nerves and from cell-to-cell by transcytosis [10,11,12] and to modulate nociceptive response secondary to peripheral nerve injury [11,13,14,15]. These effects reflect the ability of BoNTs to block the release of neurotransmitters and pro-inflammatory factors involved in pain transmission, in addition to the well-known effects on acetylcholine release at the neuromuscular junction [16]. Furthermore, BoNT/A facilitates functional recovery and accelerates the regenerative processes after peripheral nerve injury [13,15].

In the present, report we demonstrate that BoNT/A is a feasible treatment in the care of SCI and the associated symptoms, from paralysis to pain.

## 2. Results

**Figure 1:**
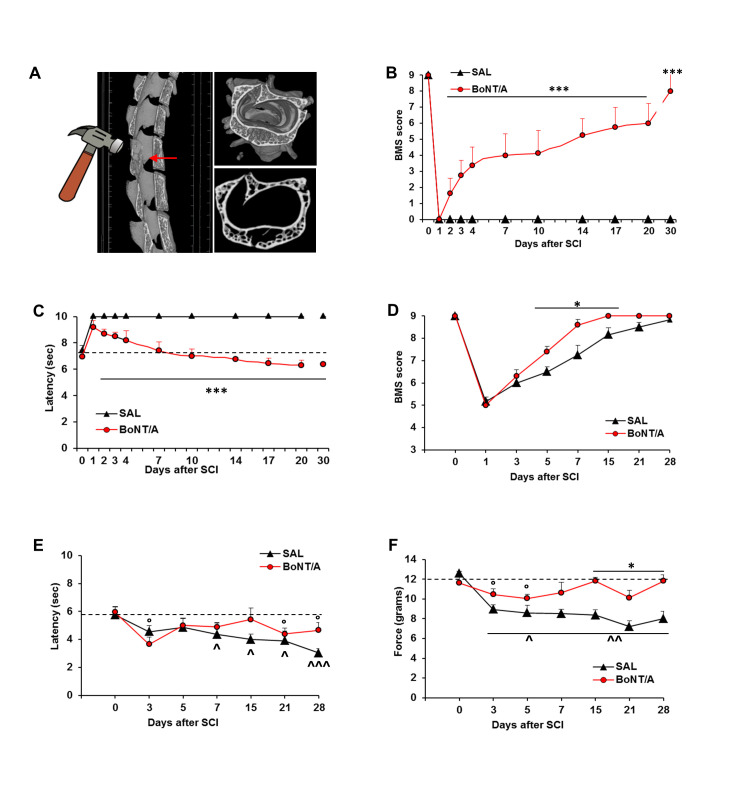
Botulinum neurotoxin type A (BoNT/A) induces recovery of motor function and restoration of spinal reflexes and sensitivity. (**A**) X-ray microtomographies showing the effect of the impact on vertebrae (T9–T11). (**B**) Basso Mouse Scale (BMS) scores as evaluation of motor function, and (**C**) tail-flick latency as measurement of thermal sensitivity in saline (SAL)- and BoNT/A-treated CD1 mice (*n* = 14/group) after the new model of severe SCI; *** *p* < 0.0001 vs. saline (Tukey–Kramer test). (**D**) BMS scores for motor function evaluation, (**E**) tail-flick latency for thermal sensitivity, and (**F**) dynamic plantar aesthesiometer test for mechanical allodynia, after moderate SCI in SAL- and BoNT/A-treated CD1 mice (*n* = 6/group). * *p* < 0.05 vs. saline; ° *p* < 0.05 vs. BoNT/A baseline (BL) (day 0); or ^ *p* < 0.05, ^^ 0.005, ^^^ 0.0001 vs. saline BL (day 0) (Tukey–Kramer test).

To assess BoNT/A efficacy in reverting motor paralysis and sensory deficits after spinal injury, we used the two animal models of SCI in CD1 (outbred) mice: (i) the new preclinical mouse model of severe contusion (Figure 1A) [8,17], to evaluate motor and sensitivity recovery, neuroprotection, and axonal regeneration; and (ii) the widely utilized model for moderate SCI [18,19], to evaluate BoNT/A effects on motor and sensitivity recovery and on NeP development.

Animals, which underwent severe or moderate SCI procedures, were spinally injected at lumbar level within 1 h from contusion with a single dose of BoNT/A (15 pg/5 µL, corresponding to the BOTOX therapeutic dose of 7.5 U/kg) or saline (as control). Since the blood–spinal cord barrier was disrupted in the impact zone and hemorrhage and ischemia occur, we chose not to administrate the toxin in the injury site (thoracic level—T9/T11), in order to avoid the BoNT/A diffusion in the hematic flux. We exploited the BoNT/A ability to be retrogradely transported to reach the damaged area, as demonstrated in the following section.

### 2.1. BoNT/A Improves Motor Control, Restores Thermal Sensitivity, and Counteracts NeP Onset in the Severe and Moderate SCI Mice

We evaluated motor recovery by using the Basso Mouse Scale (BMS) [20], thermal sensitivity restoration, and spinal reflex by tail-flick (TF) test. Following the severe trauma, a complete absence of hindlimbs’ movement was observed at day one post-SCI in both BoNT/A- and saline-treated CD1 mice (Figure 1B). At day four post-SCI, a gradual motor improvement was observed only in BoNT/A-treated CD1 mice, followed by a complete recovery of normal motor performance within 30 days in almost all subjects analyzed ($\bar{x}$= 7.75 ± SD 1.76). By contrast, saline-treated CD1 mice remained paralyzed at all time points (Figure 1B and Supplementary Materials Videos S1 and S2—example videos not representing experimental environment) (ANOVA two-way for repeated measures: treatment F_1.24_ = 106.35, *p* < 0.0001; time F_9.24_ = 58.18 *p* < 0.0001; treatment x Time F_9.216_ = 58.18, *p* < 0.0001).

Moreover, two days after SCI, BoNT/A-treated CD1 mice began to recover thermal sensitivity and completely restored TF reflex at day 20 (Figure 1C), with latencies close to the baseline (BL) values, whereas saline-treated CD1 mice showed a total absence of thermal sensitivity, reaching cutoff latency (10 sec) for the whole testing period (ANOVA two-way for repeated measures: treatment F_1.24_ = 325.84, *p* < 0.0001; time F_9.24_ = 34.86, *p* < 0.0001; treatment × time F_9.216_ = 34.86, *p* < 0.0001).

Figure 1D shows an impairment in motor function (BMS score) after a moderate trauma in both SAL and BoNT/A groups (one and three days after SCI), but from day five post-SCI, the BoNT/A-treated group showed a significant improvement in locomotion, which was early and completely recovered at day 15. Differently, locomotion in the saline-treated group was recovered only at day 28 (ANOVA two-way for repeated measures: treatment F_1.10_ = 7.479, *p* = 0.023; time F_10.7_ = 96.316, *p* < 0.0001; treatment x time F_7.63_ = 2.906, *p* = 0.0107). Moreover, saline animals developed an ongoing decrease in thermal threshold (TF test, Figure 1E), as compared to their baselines and BoNT/A-treated mice, through all test duration (ANOVA two-way for repeated measures: treatment F_1.10_ = 1.748, *p* = 0.215; time F_10.6_ = 3.528, *p* = 0.0047; treatment x time F_6.60_ = 0.96, *p* = 0.45). Finally, NeP development due to SCI was measured.

Neuropathic pain is a comorbidity frequently associated with SCI. To evaluate the BoNT/A effects in counteracting the onset of NeP, we used the moderate SCI model in which nociceptive sensitivity, different from severe SCI model, was partly maintained in the saline-treated control mice. After SCI, mice immediately reduced mechanical threshold (Figure 1F—day 3), but, while BoNT/A treatment efficiently affected NeP onset, in saline-treated mice, the decrease continued for all test duration (ANOVA for mechanical threshold: treatment F_1.10_ = 23.23 *p* = 0.0007; time F_10.6_ = 5.6 *p* = 0.0001; treatment x time interaction F_6.60_ = 3.03 *p* = 0.011).

### 2.2. Modulatory Effects of BoNT/A on Glia Scarring and Microglia Reaction

**Figure 2:**
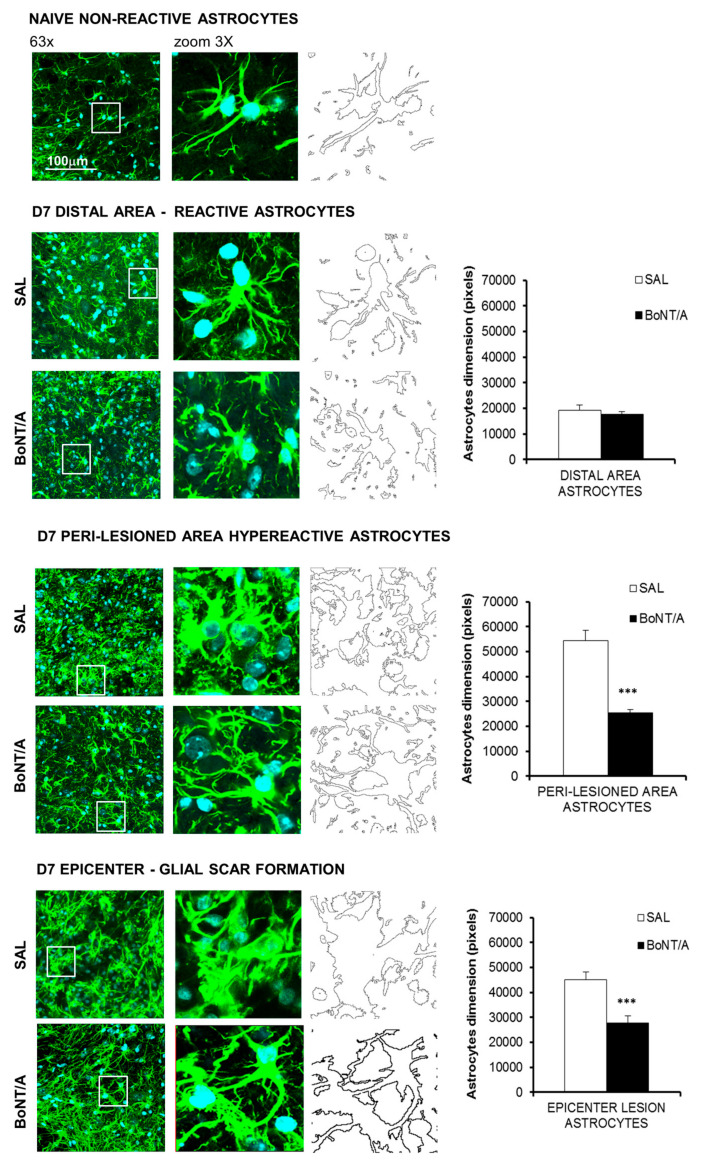
BoNT/A modulates glial cells reactivity in CD1 severe spinal cord injury (SCI) mice. Morphometric analysis of astrocytes seven days after severe SCI. Representative high magnification (63×, zoom 3) confocal immunofluorescence images taken from spinal cord of saline- and BoNT/A-treated mice. Distal Area: sections taken 5 mm rostro-caudal from peri-lesioned areas (T7–T8 or T12–T13); peri-lesioned area: sections surrounding the impact zone (T9–T11); epicenter: the core lesion (T10). Graphs show astrocytes’ dimension (pixels) digitally calculated. All analyses were performed in three different spinal areas with respect to the impact: distal, peri-lesioned and epicenter. (*n* = 7/group). *** *p* < 0.001 vs saline (Unpaired Student’s *t*-test).

One of the main obstacles to axon regeneration is due to astroglial scar around the lesion site, which acts as a physical barrier. Morphometric analysis (dimension’s evaluation—pixels) of astrocytes, carried out seven days after SCI, revealed in peri-lesioned areas hyperactive astrocytes in saline mice and only a slight reaction in BoNT/A. In addition, while in saline mice the formation of glial scar at the epicenter was strongly evident (Figure 2), astrocytes appeared hyperactive without glia scar formation in BoNT/A-treated animals. To confirm these morphological changes, each image was digitally transformed and automatically measured. Dimensional analysis showed a significant reduction in astrocytes size in both peri-lesioned (Student’s *t*-test, t_14_ = −8.29; *p* < 0.0001) and epicenter areas (Student’s *t*-test, t_17_ = −5.92; *p* < 0.0001) of BoNT/A-treated mice, revealing the modulatory ability of BoNT/A on astrogliosis. As a matter of fact, as reported in a following paragraph on cleaved SNAP25 expression, BoNT/A is able to exert its proteolytic action directly on astrocytes.

**Figure 3:**
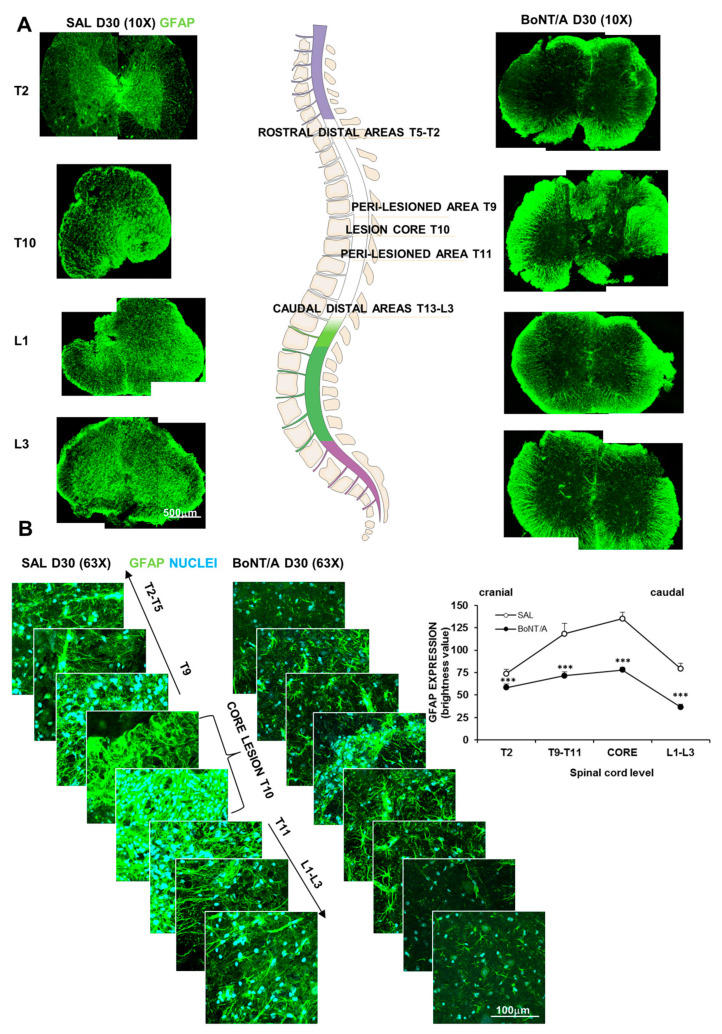
Extension of glial scar 30 days after SCI in saline- and BoNT/A-treated mice. (**A**) Representative images showing the different GFAP-stained (green) spinal levels, subsequently analyzed for the expression analysis. Spinal sections are reconstructed by combining two confocal pictures (magnification 10×) of the same slice (except for saline T10). (**B**) Analysis of GFAP expression (magnification 63×) at different levels above and below the core lesion. Graph shows fluorescence of GFAP, as quantified with RGB method, in saline-treated, compared to BoNT/A-treated, mice (five–nine slices/spinal level from three to four animals/group), Dunn-test post hoc *** *p* < 0.001, BoNT/A vs. SAL.

Glial scar formation begins early after SCI and can be considered mature after some weeks (from two to several weeks) [21]. To evaluate the BoNT/A effect on mature glial scar surrounding the core lesion and reactive astrocytes adjacent to neural tissue, we analyzed glial fibrillary acidic protein (GFAP) expression 30 days after SCI in spinal sections at different levels: from the core lesion (T10) toward peri-lesioned (T9–T11) and distal (rostral: T2–T5 or caudal: L1–L3) areas (Figure 3A). As expected, analysis of GFAP fluorescence (Figure 3B) evidenced a different pattern of expression, depending on the areas considered: areas more distant from the impact zone presented a lesser degree of astrocytes activation. However, GFAP in all areas of spinal cords was markedly enhanced in saline-treated, compared to BoNT/A-treated mice (Kruskal–Wallis test, H_7_ = 37,523 *p* < 0.0001).

**Figure 4:**
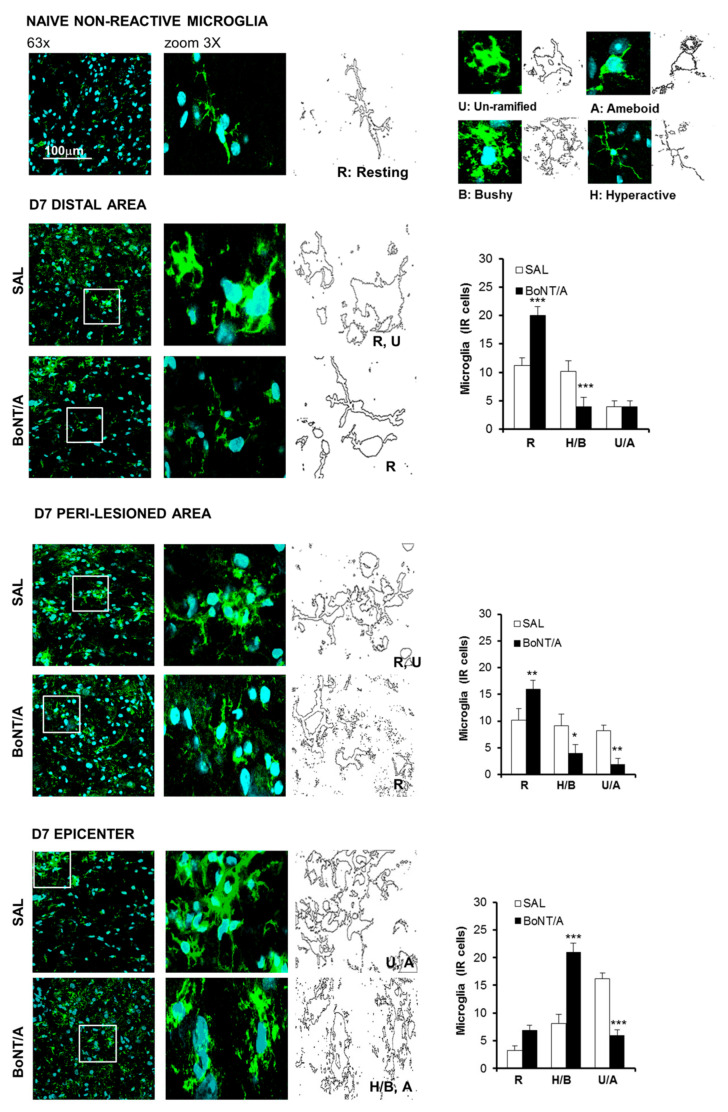
BoNT/A modulates glial cells reactivity in CD1 severe SCI mice. Morphometric analysis of microglia seven days after severe SCI. Representative high-magnification (63×, zoom 3) confocal immunofluorescence images taken from spinal cord of saline- and BoNT/A-treated mice. Distal Area: sections taken 5 mm rostro-caudal from peri-lesioned areas (T7–T8 or T12–T13); peri-lesioned area: sections surrounding the impact zone (T9–T11); epicenter: the core lesion (T10). Graphs show the count of different microglia phenotypes (R, resting/ramified; H/B, hyperactive/bushy; and U/A, un-ramified/ameboid). All analyses were performed in three different spinal areas, with respect to the impact: distal, peri-lesioned, and epicenter. (*n* = 7/group). * *p* < 0.01, ** *p* < 0.01, *** *p* < 0.001 vs. saline (Tukey–Kramer Test).

After SCI, also microglia participate in neuroinflammation; these cells, seven days after SCI, appeared in a hyperactive state (Figure 4). Since microglia assumes several shapes depending on its activation status, we identified and counted the different phenotypes present and distributed in the analyzed areas: resting (R), hypertrophic/bushy (H/B), and un-ramified/amoeboid (U/A) microglia. BoNT/A significantly increased R and decreased H/B states in distal and peri-lesioned areas (ANOVA, F_5.36_ = 38.482; *p* < 0.0001; F_5.36_ = 14.824; *p* < 0.0001), while an increase of H/B and a decrease of U/A phenotypes were observed at the epicenter (ANOVA, F_5.36_ = 32.123; *p* < 0.0001), supporting the idea of a phagocytic activity, which may contribute to maintenance of homeostasis.

### 2.3. Protective Effects of BoNT/A on Lipid and Glycemic Profiles, Cell Death, and Remyelination

During acute SCI, low levels of plasma lipids and hyperglycemia represent detrimental factors that impede functional improvement in mice and humans and correlate with a more serious trauma and more severe motor deficits, predicting more intensive pain and a worst prognosis [22,23,24].

**Figure 5:**
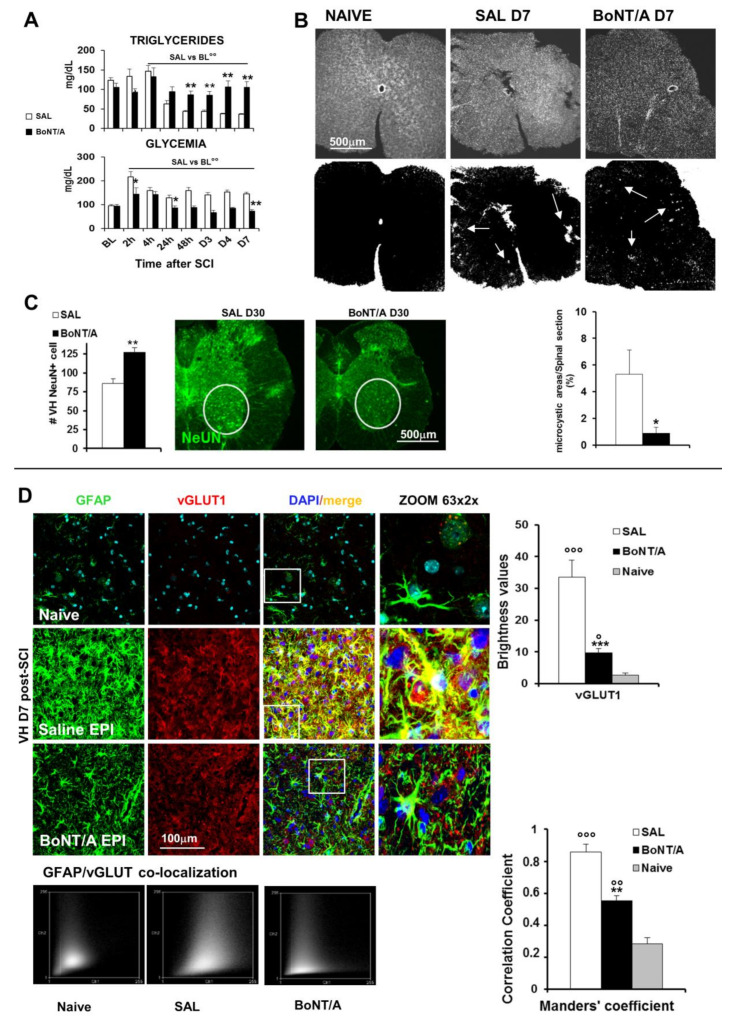
BoNT/A treatment on prognostic metabolic parameters, neurodegeneration, and excitotoxicity in severe SCI mice. (**A**) Triglycerides and glucose blood levels in saline- and BoNT/A-treated mice, as function of time after SCI (*n* = 11/group). °° *p* < 0.01 SAL vs. BL (baseline); * *p* < 0.05, ** *p* < 0.01 BoNT/A vs. SAL (Tukey–Kramer Test). (**B**) Representative images of *Microcystis* (arrows) in spinal cord sections (T9–T11, magnification 10×) immunostained for DAPI (nuclei) from saline- and BoNT/A-treated mice seven days after SCI and converted in binary images to evidence damaged areas. In graph: microcystic degeneration is quantified as percentage ratio between overall cavity dimension and spinal section area measured in pixels (cavity/spinal section* 100 = % of total damaged area) (*n* = 7/group), Student’s *t*-test, * *p* < 0.05 vs. SAL. (**C**) Loss of motor neurons (magnification 10×), caudal to the lesion (L2), 30 days after SCI (*n* = 9/group, Student’s *t*-test); ** *p* < 0.01 vs. SAL. (**D**) Representative images (magnification 63×, zoom 2) showing the expression of astrocytes (GFAP; green), vGLUT1 (red) and their colocalization in saline- and BoNT/A-treated mice, 7 days after SCI, at the epicentre (EPI) (*n* = 6/group). Nuclei are stained with DAPI (blue). Graphs show fluorescence of vGLUT1, as quantified with RGB method, in saline- and BoNT/A-treated compared to Naive mice, and its colocalization with GFAP, analysed by Manders correlation coefficient, represented also by scatter plot. ° *p* < 0.05, °° *p* < 0.01, °°° *p* < 0.001 vs naïve; ** *p* < 0.01, *** *p* < 0.001, BoNT/A vs SAL.

In saline-treated mice, as also reported for human subjects, triglycerides blood levels decreased, starting from 24 h after SCI, while in BoNT/A-treated mice, levels comparable to the baseline were maintained (Figure 5A, ANOVA two-way for repeated measures: time F_7.20_ = 17.08 *p* < 0.0001; treatment x time F_7.140_ = 10.47, *p* < 0.0001). Moreover, when glucose blood levels were examined in the saline group, a hyperglycemic status starting 2 h after SCI and lasting for the entire testing period (day seven) was observed, while BoNT/A treatment preserved a normoglycemic level (Figure 5A, ANOVA two-way for repeated measures: treatment F_1.20_ = 33.15, *p* < 0.0001; time F_7.20_ = 12.7 *p* < 0.0001; treatment x time F_7.140_ = 3.309, *p* < 0.001).

Loss of nervous tissue in the injured area (thoracic level) begins early after trauma (seven days), with the microcystic formation, followed by the involvement of neurons also distally (caudal) to the lesion (day 30). We observed a severe degeneration at day seven post-SCI, at the thoracic level, where spinal sections from saline-treated mice showed a higher presence of *Microcystis* in the lesioned area in comparison with those derived from BoNT/A (Student’s *t*-test, t_12_ = −2.197; *p* = 0.0484—*Microcystis* dimension is evaluated in pixels in respect to the total spinal section area dimension, Figure 5B). Thirty days after SCI, BoNT/A treatment was also able to preserve lumbar motoneurons and to promote their survival (Figure 5C), while in saline-treated animals, they were compromised and reduced in number (Student’s *t*-test, t_16_ = −3.401; *p* = 0.0036).

To investigate mechanisms by which BoNT/A exerted its neuroprotective and anti-inflammatory effects, we analyzed the vesicular transporter of glutamate 1 (vGLUT1) and its expression in astrocytes (Figure 5D) seven days after SCI, being glutamate one of the major responsible of excitotoxicity-induced cell death [25]. In epicenter zone (EPI, area of impact), an impressive presence of vGLUT1 (see brightness value in graph) and a strong colocalization with the astrocyte marker GFAP in saline mice was detected (ANOVA, F_2.15_ = 10.634; *p* = 0.0013). This colocalization was also put in evidence by the analysis of the Manders’ correlation coefficient that resulted in being significantly higher than in BoNT/A animals (see graph, ANOVA, F_2.15_ = 84.539; *p* < 0.0001), suggesting that the reduction of vGLUT in BoNT/A-treated mice may be ascribable to a reduction of glutamate release.

**Figure 6:**
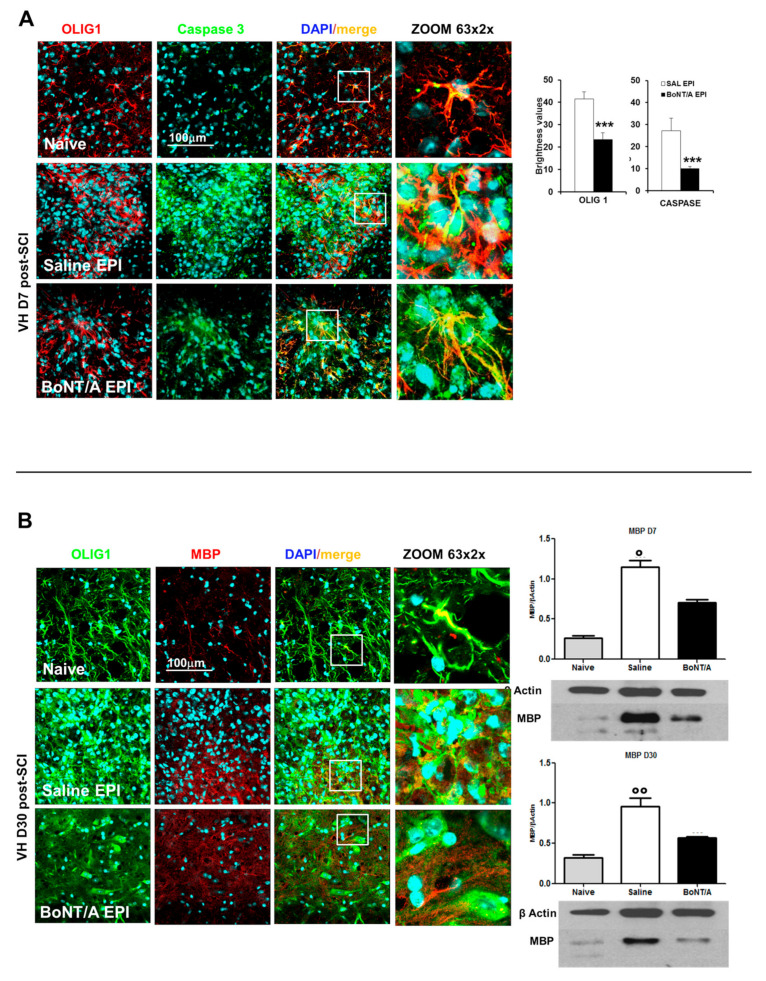
BoNT/A induces neuroprotective effects in CD1 severe SCI mice. (**A**) Representative images showing the expression of oligodendrocytes (OLIG1; red), Caspase-3 (green), and their colocalization in naïve, saline- and BoNT/A-treated mice, seven days after SCI (*n* = 9/group; magnification 63×, zoom 2). Graphs show fluorescence of OLIG1 and Caspase, as quantified with RGB method, at the epicenter (EPI). *** *p* < 0.001, BoNT/A vs. SAL. (**B**) Representative images showing the expression of oligodendrocytes (OLIG1; green), MBP (red), and their colocalization in naïve, saline- and BoNT/A-treated mice, seven days after SCI (Magnification 63×, zoom 2×). Nuclei are stained with DAPI (blue). Protein quantification (WB) of MBP in Naïve and saline- or BoNT/A-treated mice, 7 and 30 days after SCI, is reported in graph (*n* = 3/group – D7; *n* = 4/group—D30). ° *p* < 0.05; °° *p* < 0.001 vs. Naïve, Dunn’s multiple comparison Test.

Mature oligodendrocytes, the only myelin-forming cells within the CNS, are highly susceptible to SCI and undergo apoptosis [26]. Seven days after injury, in the epicenter of spinal impact, we observed in saline mice (Figure 6A) a massive expression of Caspase 3 (an apoptotic marker) partially co-localizing with oligodendrocyte (OLIG1). On the other hand, BoNT/A treatment significantly affected Caspase 3 expression (Student’s *t*-test, t_16_ = −2.953 *p* = 0.0093), suggesting a protective effect of the toxin, although a certain co-localization with OLIG1 was observable. A major expression of OLIG1 was even detected both in epicenter (Student’s *t*-test, t_16_ = −4059 *p* < 0.0001) and in peri-lesioned area of injured spinal cord of saline-treated mice (Figure S1), even if the increase in expression can be attributable to different morphology of the oligodendrocytes, which in saline-treated animals appeared deranged and in BoNT/A more similar to naïve mice. Moreover, in PERI area, stripe-like structures of oligodendrocytes were visible in saline spinal cords, while a non-reactive condition was present in BoNT/A-treated mice (Figure S1).

After SCI, the spared tissue exhibits abnormal myelination, associated with reduced or blocked axonal conduction. It was reported that a major constituent of CNS myelin, myelin basic protein (MBP), is produced as “reactive” response of surviving oligodendrocytes and peaked around day eight, in the attempt to induce repair mechanisms [27]. We found an increase in MBP in our SCI model seven days after injury in saline-treated mice (Figure 6B, H_3_ = 7.2 *p* = 0.0273), evident also at day 30 (H_3_ = 9.846 *p* = 0.0073), while in BoNT/A treated animals MBP approximated the naïve levels, supporting a reduced reaction of oligodendrocytes, probably due to a minor degree of degeneration, as also illustrated in representative confocal images from spinal tissue collected after 30 days from contusion, stained for oligodendrocytes (OLIG1) and MBP.

### 2.4. Effects of BoNT/A on Stem Cells Stimulation and 3D Spinal-Cord Reconstruction

The region surrounding the central canal is considered a potential stem cell niche, since these cells react to injury by proliferating and migrating toward the lesion and being a source for repair [28]. Due to the impressive ability of BoNT/A to induce motor recovery, we investigated the possibility that it promoted stem cells proliferation.

**Figure 7:**
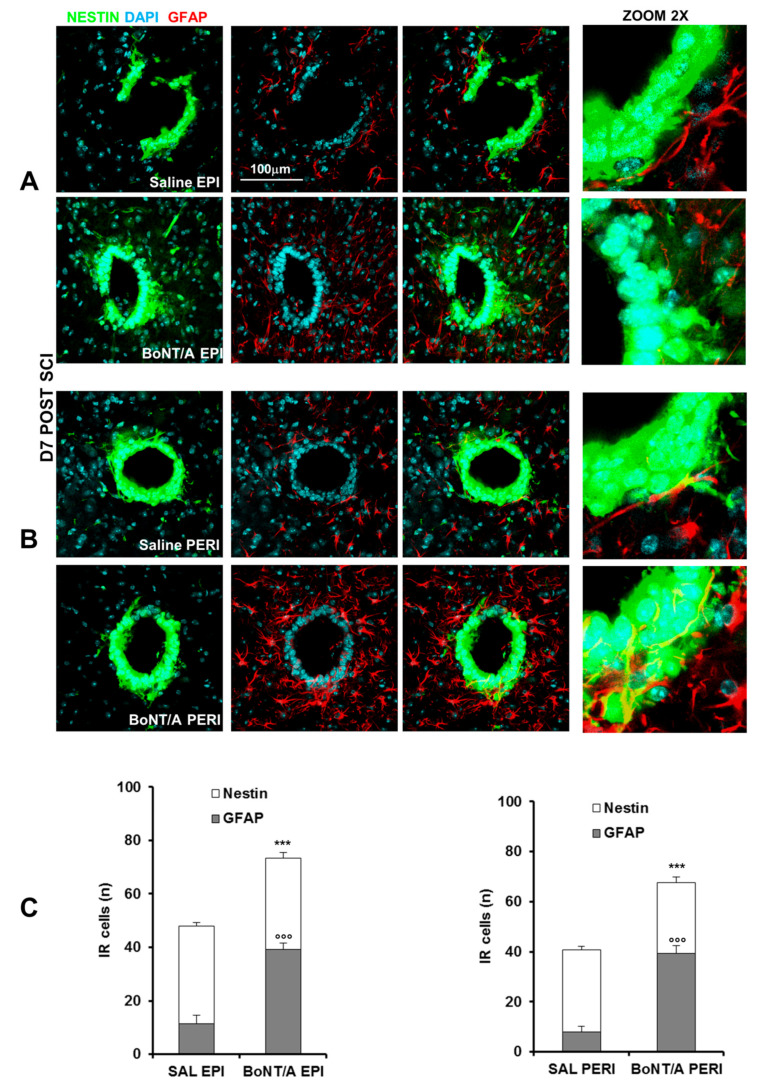
BoNT/A promotes stem cells proliferation in spinal cord of C57BL/6N nestin-GFP (green fluorescent protein) severe SCI mice. Representative images showing nestin-GFP cells (green), GFAP (red), and their colocalization in central canal of spinal cord, both at epicenter (EPI) (**A**) and in peri-lesioned area (PERI) (**B**) of saline- and BoNT/A-treated mice, seven days after SCI (magnification 63×, zoom 2). Nuclei are stained with DAPI (blue). (**C**) Bar graphs show the quantification of total nestin cells and nestin/GFAP-positive cells in saline- and BoNT/A-treated SCI mice. *** *p* < 0.001, °°° *p* < 0.001 BoNT/A vs. SAL (*n* = 6/group, Student’s *t*- test).

To this aim, we took advantage from transgenic nestin-GFP (green fluorescent protein) mice. After contusion we counted nestin-GFP cells (stem cells) colocalized with GFAP in central canal of spinal cord, at epicenter (Figure 7A) and in peri-lesioned (Figure 7B) area. Graphs show (Figure 7C) a great enhancement of nestin cells (Student’s *t*-test, t_10_ = 5.06 *p* = 0.0005) and a significant increase of GFAP positive cells (Student’s *t*-test, t_10_ = 10.371 *p* < 0.0001) in epicenter (Figure 7A) and in peri-lesioned area (Student’s *t*-test, nestin peri: t_10_ = 7.114 *p* < 0.0001 and GFAP peri: t_10_ = 11.18 *p* < 0.0001) of BoNT/A-treated mice (Figure 7B).

**Figure 8:**
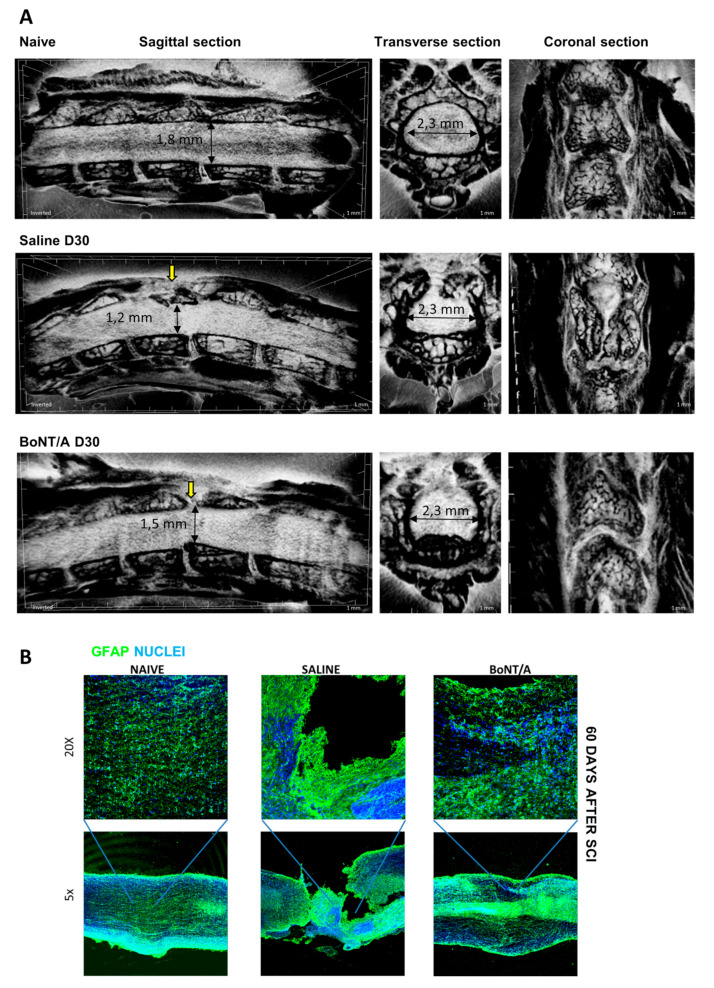
Nervous tissue recovery. (**A**) Representative images of MicroCT 3D-video reconstruction of spinal cord of naïve, saline-treated, and BoNT/A- treated mice 30 days after trauma. Yellow arrows indicate the impact zone. (**B**) Representative image of immunostained longitudinal spinal cords section of naïve, saline-treated, and BoNT/A-treated animals, 60 days after SCI. GFAP (green) and nuclei (blue).

After having demonstrated that BoNT/A was able to restore motor function acting on different systems, here we show the spinal cord (micro-CT) reconstruction 30 days after injury (Video S3—naïve, Video S4—saline, and Video S5—BoNT/A; Figure 8A) and immunostaining of longitudinal section of spinal cord 60 days after trauma (Figure 8B). As well distinguishable in Figure 8A, 30 days after SCI, the spinal cord of saline-treated mice showed a remarkable loss of tissue in comparison with the spinal cord of naïve animals (from 1.8 mm of dorsal/ventral diameter, to 1.2 mm, about 35% less), still present after two months from injury (Figure 8B) in the impact zone. On the contrary, treatment with BoNT/A was able to facilitate nervous tissue regeneration (1.5 mm of diameter), as also shown in immunostained tissue (Figure 8B). These differences are particularly evident in the 3D videos during deconvolution for transverse section, in which the injured area of spinal cord in saline-treated mice (Video S4) appears completely deformed and gray matter only scarcely perceptible in comparison with naïve (Video S3) and BoNT/A-treated mice (Video S5).

### 2.5. Muscle Atrophy Recovery after BoNT/A Treatment as Consequence of Motor Neurons Reconnection

SCI induces loss of motoneurons, leading to muscular atrophy due to activation of catabolic machinery [29]. Therefore, recovery of muscle tone and functionality implies a motor neuron–muscle reconnection. We analyzed quadriceps muscle, to indirectly evaluate if BoNT/A was able to reactivate neuromuscular connection.

**Figure 9:**
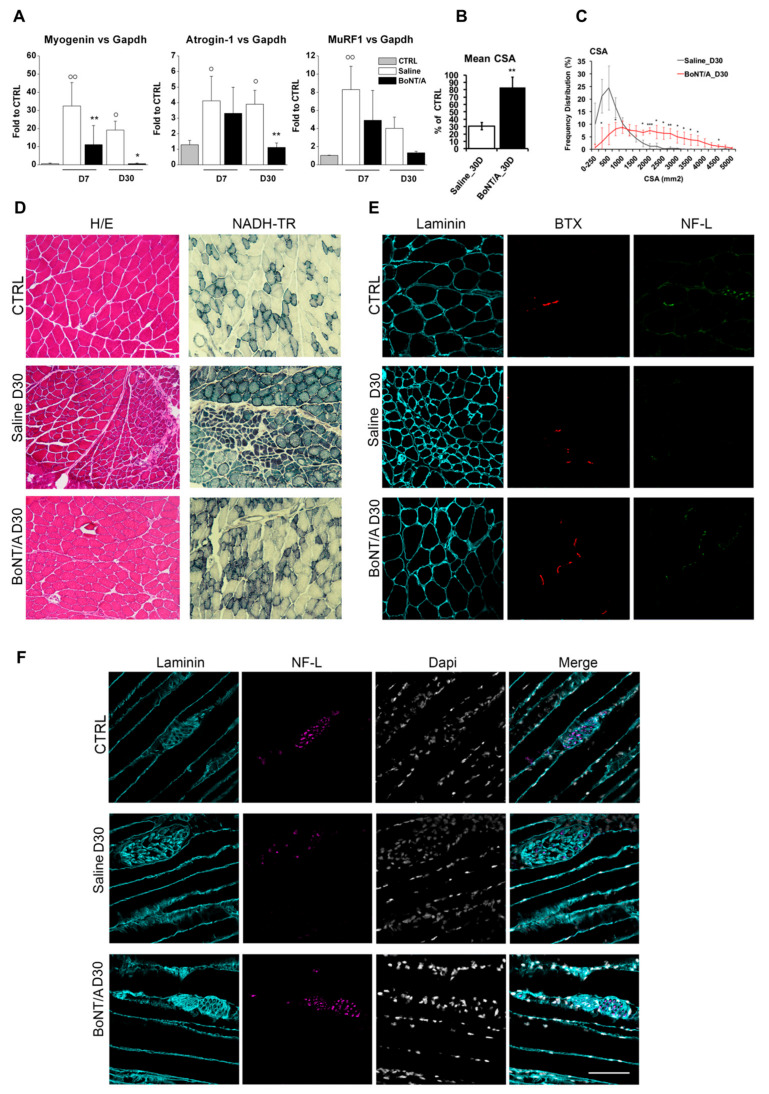
Muscle atrophy after SCI. (**A**) Relative expression of Myogenin, Atrogin-1, and MuRF1 in quadriceps muscle isolated from saline- and BoNT/A-treated mice, 7 and 30 days after SCI (*n* > 3). * *p* < 0.05; ** *p* < 0.01 (**B**) The graph shows mean cross-sectional area (CSA) of saline- and BoNT/A-treated quadriceps muscle fibers 30 days after SCI. ** *p* < 0.01; by student *t* Test (*n* = 3 saline and 4 BoNT/A). (**C**) Frequency distribution of quadriceps muscle fibers CSA in saline or BoNT/A-treated mice 30 days after SCI. The line graph represents relative frequencies as percentage. * *p* < 0.05, ** *p* < 0.01, *** *p* < 0.001 vs SAL; ° *p* < 0.05, °° *p* < 0.01 vs CTRL; by student T-test (*n* = 3 saline and 4 BoNT/A). (**D**) Representative images of Hematoxylin/Eosin (left) and NADH–TR (right) staining of naïve, saline and BoNT/A muscle cryosections, 30 days after SCI (bar = 200 μm). (**E**) Representative images of Laminin (cyan), BTX (red), and Neurofilament-L (green) staining of naïve, saline, and BoNT/A muscle cryosections, 30 days after SCI (bar = 50 μm). (**F**) Attenuated neuronal loss in skeletal muscle of BoNT/A-treated SCI mice. Representative images of Laminin (cyan) and Neurofilament-L (purple) staining in naïve, saline, and BoNT/A muscle longitudinal cryosections, 30 days after SCI (bar = 100 μm).

Figure 9A shows that, seven days after SCI, muscle-specific ubiquitin ligases Atrogin-1 and MuRF1 and their upstream regulator, Myogenin [30], were strongly induced in muscles of saline-treated mice, but not in BoNT/A-treated mice. Likewise, 30 days post-SCI, Myogenin, Atrogin-1, and MuRF-1 continued to be expressed only in muscles of saline-treated mice (ANOVA one-way; Myogenin F_4.19_ = 10.658 *p* < 0.001; Atrogin F_4.20_ = 7.692 *p* < 0.001; MuRF1 F_4.20_ = 10.167 *p* < 0.001). Consistently, BoNT/A treatment attenuated SCI-induced reduction of cross-sectional area (CSA) (Figure 9B,C) (Student’s *t*-test t_5_ = 6.209 *p* = 0.0016). This finding suggests that catabolic genes are induced by pathological neurotransmission, rather than by interruption of neuromuscular junction NMJ communication to muscles, upon SCI.

Hematoxylin–Eosin (H/E) and nicotinamide adenosine dehydrogenase–tetrazolium reductase (NADH–TR) staining revealed that the diffuse fibers atrophy and extensive muscle fibers degeneration observed in saline-treated mice were significantly attenuated by BoNT/A administration (Figure 9D). Moreover, while in control (CTRL) mice, SCI resulted in loss of muscle innervation, BoNT/A treatment preserved neuron-muscle connection, as revealed by the double staining with anti-Bungarotoxin (BTX) and Neurofilament-L (NF-L) (Figure 9E), as well as the anatomical integrity of nerve structures in skeletal muscle (Figure 9F).

### 2.6. Long-Lasting Proteolytic Action of BoNT/A Affects Astrocytes and Neurons of Spinal Cord but Not Brain

**Figure 10:**
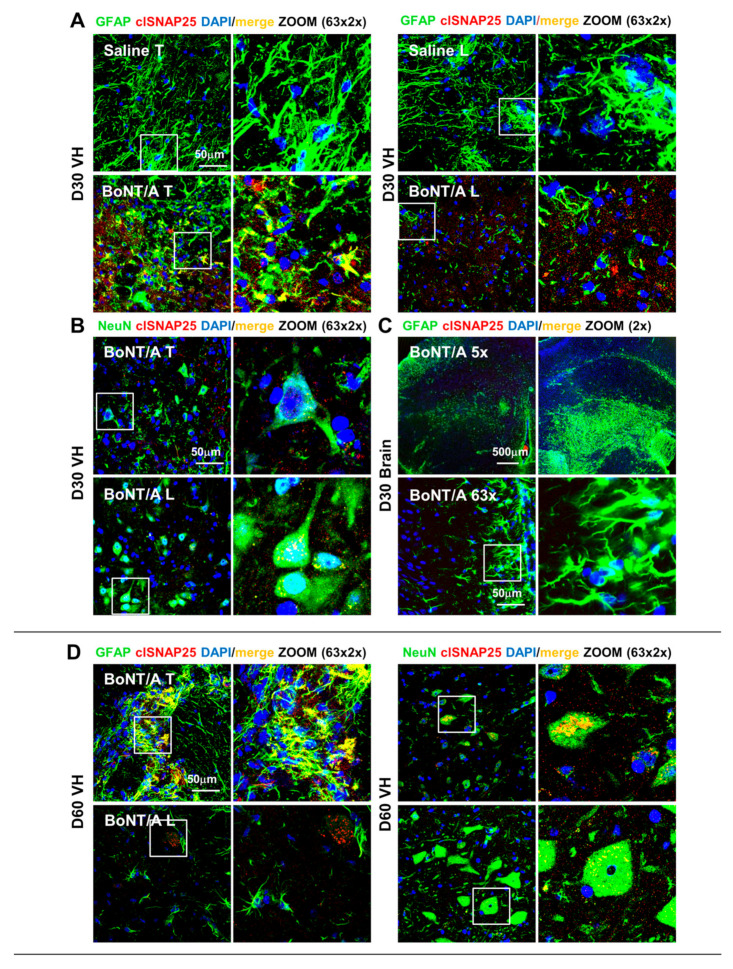
Localization of cleaved-SNAP25 in spinal cord and brain of SCI mice. (**A**) Representative images showing the expression of cl-SNAP25 in spinal ventral horns (VH) region, close (T) to and distant (L) from the impact zone in saline- and BoNT/A-treated mice, 30 days after SCI. The cleaved(cl)-SNAP25 (red) is colocalized with GFAP (green; marker of astrocytes) and with DAPI (blue; marker of nuclei). White squares indicate regions where ZOOM (63×, zoom 2×) was taken. (**B**) Confocal immunofluorescence images showing the expression of cl-SNAP25 in spinal VH region close (T) and distant (L) from the impact zone in BoNT/A-treated mice, 30 days after SCI. The cl-SNAP25 (red) is colocalized with NeuN (green; marker of neurons) and DAPI (blue). (**C**) Confocal immunofluorescence images showing the expression of cl-SNAP25 colocalized with GFAP in spinal VH region close (T) and distant (L) from SCI impact zone of BoNT/A-treated mice, 60 days after SCI. (**D**) Confocal immunofluorescence images showing the expression of cl-SNAP25 colocalized with GFAP (left panels) or NeuN (right panels) in spinal VH region close (T) and distant (L) from SCI impact zone of BoNT/A-treated mice, 60 days after SCI. The cl-SNAP25 (red) is colocalized with GFAP (green) or NeuN (green) and with DAPI (blue). White squares indicate regions where ZOOM (63×, zoom 2×) was taken.

Considering the very low amount of toxin used (therapeutic concentration), below the range of detectability, the only method to detect the presence of BoNT/A, as well as its duration and diffusion from the injection site (lumbar), is to examine the cleaved (cl-) SNAP25 expression [31], which represents the toxins’ product. In spinal ventral horns, immunofluorescence (IF) analysis revealed cl-SNAP25 expression both at the injection site (lumbar level, L4/L5) and at thoracic level (T9/T11, impact zone) in BoNT/A mice, demonstrating the toxin retrograde transport; a complete absence in saline-treated animals was observed. In particular, we observed cl-SNAP25 immunoreaction both on astrocytes (co-localization with GFAP, Figure 10A) and neurons (co-staining with NeuN, Figure 10B) 30 days after SCI at close (T) and distant (L) level; of relevance for its therapeutic value, it is the evidence that cl-SNAP25 is still detectable after two months from injection (Figure 10C,D), indicating the ability of a single administration to act over 60 days from SCI. Finally, no presence of cl-SNAP25 was detectable in the whole brain (representative example in Figure 10C).

## 3. Discussion

Among bacteria toxins, BoNTs represent a notable successful translation from basic science to clinical applications, resulting in useful therapeutic agents for several human diseases. The present study extends the translational potential of BoNT/A to one of the major challenges of medicine, the care of SCI, which causes loss of spinal cord tissue and consequently loss of motor, sensory and autonomic function. Our results are particularly relevant, considering that, according to the World Health Organization (2013), between 250,000 and 500,000 people suffer a SCI every year, living with devastating disabilities.

Here we demonstrate the great pro-regenerative and therapeutic potential of BoNT/A to recover motor output and sensitivity, in a preclinical animal model able to mimic human-like features of SCI [8,17] and to prevent NeP development in a model of moderate SCI.

While initial damage induced by SCI is directly ascribed to the trauma (e.g., hemorrhage, membrane disruption, and vascular damage), the final lesion is far greater than that identifiable in the first few hours after the injury. The impairments are permanent because intrinsic and extrinsic mechanisms act in parallel to impede repair events and restoration of the damaged axonal circuits [32], continuously changing SCI pathophysiology and making therapeutic intervention particularly difficult. The spread of the damage is thought to be because of the detrimental events activation, leading to cellular dysfunction and death. The cascade of these injury-induced destructive events (summarized in Table 1) is defined as secondary injury. We showed that BoNT/A administration positively affects many of these adverse phenomena. In particular, BoNT/A has proven able to interfere with the glial scar formation and microglia activation. These two events, while necessary to curbing the damage [33,34], are the most responsible for the impenetrable environment that hampers the axonal sprouting and reconnection [35,36,37]. Thus, BoNT/A, modulating the neuroglia SCI response, allows a gradual progression toward the recovery.

**Figure 11:**
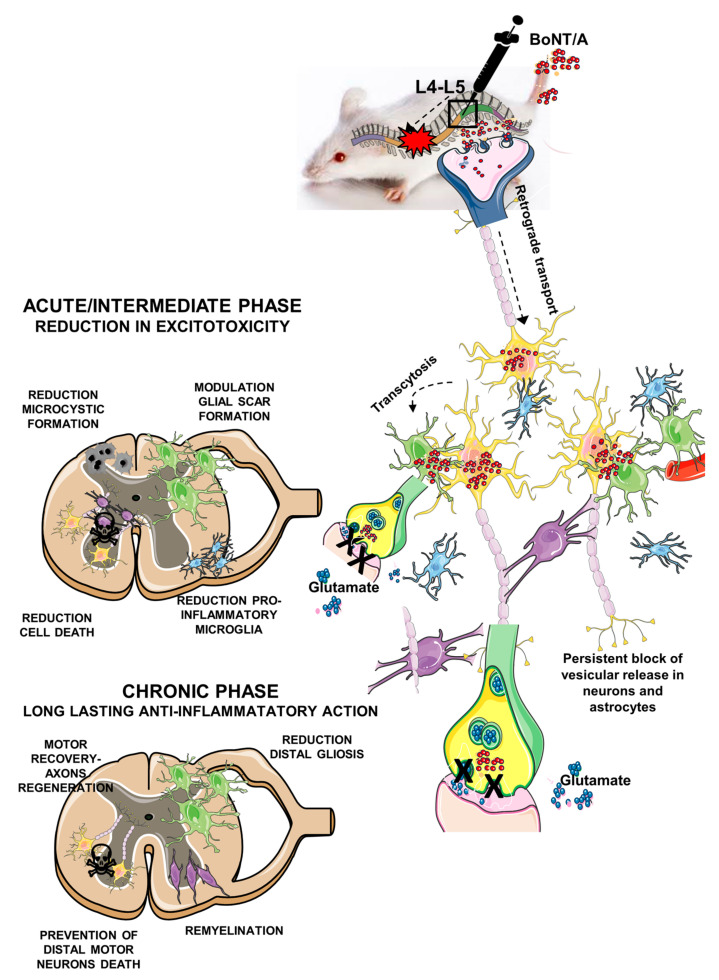
Schematic representation of proposed mechanism of action of BoNT/A. BoNT/A (red dots) is spinally administered within 1 h from spinal trauma in lumbar zone. BoNT/A enters in neurons by binding to the synaptic vesicle transmembrane protein SV2 (the BoNT/A protein receptor) and by transcytosis (dashed curved arrow) in astrocytes. From inoculation site, it is retrogradely transported (dashed arrow) along axons, reaching the impact zone and distal areas. BoNT/A blocks vesicular glutamate (blue dots) release, reducing excitotoxicity, and thus influencing and reducing both pro-apoptotic phenomena and inflammatory events in the acute/intermediate phase and favoring a hospitable environment for axonal regrowth. Since BoNT/A has a long-lasting proteolytic activity (up to 60 days), persistent modulation of pro-inflammatory agents and neuroprotection determine a complete recovery of motor function and neural re-connection spinal cord/muscles. (Thanks to Servier Medical Art to share and for making scientific cartoons available as an open source for the community.)

We previously showed [11], and here confirmed, that BoNT/A is able to enter and to act into the astrocytes, as evidenced by the colocalization of GFAP with cl-SNAP25. In this study, BoNT/A exerted a modulatory action on astrocytes, reducing their hyperreactivity and modulating glial scar formation in the lesion epicenter; moreover, the neurotoxin avoided astrocytes’ secondary reaction in peri-lesioned areas. This BoNT/A modulatory effect was also detectable in microglia response, which shifts from a pro-inflammatory to a phagocytic phenotype, thus indicating homeostasis promotion. The present results are in line with our previous studies in murine models of neuropathic and inflammatory pain [37,38]. A schematic representation of the proposed mechanism for the action of BoNT/A in SCI repair is reported in Figure 11, where the complex phenomena associated with SCI are summarized.

In the last years, a great deal of attention has been focused on the possible metabolic changes occurring after neurological trauma. The importance of these changes appears to be related to the severity of damage. In clinical studies, after SCI and brain injuries, low levels of serum lipids are related to more serious damage, a worst prognosis, and enhanced pain perception [22,24]. Moreover, trauma leads to hyperglycemia during the acute phase of injury, and this is recognized as a detrimental factor that impairs functional improvement in mice and humans [23,38]. Since our intention is also to provide information on the therapeutic power of BoNT/A, we evaluated these prognostic metabolic parameters. We found alterations similar to those observed in humans in saline-treated mice, confirming a poor prognosis, while in BoNT/A-treated mice, we detected normal lipid and glucose levels, indices of positive prognosis.

One of the biochemical consequences triggered by SCI is the increased extracellular glutamate concentration to neurotoxic levels [25]. Under normal conditions, vGlut1 rapidly removes extracellular glutamate from the synaptic cleft, preventing its accumulation. We observed a strong decrease of vGlut1 expression after BoNT/A administration, thus supporting a reduction of glutamate release. A limited excitotoxicity may be an important consequence of BoNT/A action at the synaptic level, where the toxin interfering with the SNARE complex and exocytosis inhibits glutamate release [11,13,14,15,16]. In fact, excitotoxicity promotes apoptosis in neurons and oligodendrocytes, and BoNT/A treatment dramatically reduced it, evidencing its neuroprotective ability. As a consequence, the oligodendrocytes’ survival was increased and counteracted the progressive demyelination.

Because we observed that BoNT/A was able to reduce tissue damage and neuronal loss and to promote motoneurons’ survival, it was stimulating to determine its effects on stem cells proliferation. Currently, it is widely demonstrated that, in mammalian mature CNS, in stem cells niches and zones of proliferating precursor cells, neural precursor cells (NPCs) exist [39]. They are multipotent and involved in self-renewal, and they are able to differentiate into neurons, astroglia, and oligodendrocytes. It is reported that NPCs are nestin^+^ and highly expressed after SCI, mainly in dorsal horns of the spinal cord and in central canal [40,41]. We used GFP-nestin engineered mice, and we found nestin+ cells strongly expressed in the central canal seven days after SCI; BoNT/A treatment induced in this area an enhancement of nestin+ cells’ (undifferentiated stem cells) total number and of those colocalized with both GFAP [42]. Because it has been demonstrated that inflammation affects stem cells’ survival, self-renewal, migration, and differentiation [43,44], the modulatory effect on inflammatory processes induced by BoNT/A may indirectly facilitate NPCs proliferation. The increase of these cells after BoNT/A is an interesting starting point for future experiments aimed to (i) better clarify the final fate of nestin+ cells, extending our investigations besides the central canal, and (ii) comprehend the role of the toxin in the complex interaction between proliferation and inflammation. The possibility that a pharmacological treatment can stimulate endogenous stem cells in the adult spinal cord and promote functional recovery paves the way for developing new therapeutic strategy.

Finally, 3D spinal cord reconstruction clearly demonstrated that, in saline-treated mice, a great portion of nervous tissue was definitively lost after 30 days after SCI, while in BoNT/A-treated mice, a less damaged spinal cord was observable.

After SCI, important changes occur to the musculoskeletal system, due to the breakdown of communication between CNS and parts of body below the injury site, changes that cause the partial or total overall functions loss and detrimental comorbidities, including NeP [45]. SCI patients are known to experience bone loss and muscle atrophy [46]. Muscles in SCI individuals present morphological and contractile changes characterized by decreased dimension and fat infiltration in muscular fibers.

When the motor neuron–muscle connection is restored, the muscle reactivates its function. In fact, the surprising motor and sensory functions’ recovery in BoNT/A-treated mice was accompanied by gene expression modulation involved in muscular atrophy, such as Myogenin or Atrogin-1 and MuRF1 (two muscle-specific E3 ubiquitin ligases) [30], which were increased transcriptionally in saline mice’s quadriceps muscle. This indicates an atrophy-inducing condition 7 and 30 days after SCI. Otherwise, BoNT/A treatment prevented paralysis-induced hindlimbs muscle atrophy. Moreover, histological analysis showed a remarkable restoration of muscle fibers in 30-day BoNT/A-treated mice.

We think that the long-term duration of action, a peculiarity of BoNT/A (about two to six months in patients) at concentrations as low as fM or pM, confirmed also in our mouse model, confers to this neurotoxin an advantage in comparison with the common drugs that need to be continuously administered to maintain a therapeutic effect.

Altogether, our findings reveal the extraordinary ability of BoNT/A, which had never been demonstrated before, in neuroprotection and promotion of CNS regeneration, in a clinically relevant animal model of SCI. Although the comprehension of molecular events responsible for the regenerative ability of BoNT/A needs to be deeply elucidated, and further investigations are requested, our study opens a new scenario in the therapy and care of spinal lesions and encourages clinical translation, with the pharmacology, safety, and toxicology of BoNT/A being already known.

## 4. Materials and Methods

### 4.1. Animals

Three-month-old CD1 female mice (Charles River Laboratories, Como, Italy) and 8–10-week-old and 3-month-old C57BL/6N nestin-GFP female mice (kindly provided by G. Enikolopov, Cold Spring Harbor Laboratory, Cold Spring Harbor, NY, USA, and generously offered by Stefano Farioli Vecchioli, CNR-IBBC, Roma, Italy) were used. In these transgenic mice, the neural stem cells of the embryonic and adult brain are marked by the expression of green fluorescent protein (GFP) [47]. The fluorescent signal from the transgene was so strong that transgenic P1-3 postnatal mice were routinely determined by using a fluorescent lamp.

Animals were housed in standard transparent plastic cages, in groups of 4 per cage, lined with sawdust, under a standard 12/12 h light/dark cycle (7:00 a.m./7:00 p.m.), with food and water available ad libitum. Thirty minutes before surgery, the mice were placed in the experimental room, and they were randomly assigned to the different experimental groups. A first investigator performed the surgery and randomly administered the treatment, assigning an ID number for treatment/mouse. Testing was performed from blind investigators as for treatment groups for all experiment duration. At the end of the statistical analysis, the correspondence (ID number/treatment) was revealed. Care and handling of the animals were in accordance with the guidelines of the Committee for Research and Ethical Issues of IASP [48], and all in vivo procedures were approved by the Italian Ministry of Health (PR33/2014, 6 November 2014; PR122/2019, 10 February 2019) on the use of animal for research. The number of animals utilized for each experiment and group is reported in the figure legend.

### 4.2. Surgery

We utilized two different models to perform SCI. On the basis of our previous paper [8], we tested BoNT/A efficacy in CD1 mice subjected to severe SCI in the new model without laminectomy. In regard to the moderate contusion model, SCI was performed with laminectomy.

#### 4.2.1. Severe Trauma in CD1 Mice

To perform the severe spinal cord injury (SCI) in the CD1 and nestin-GFP mice, we used the new model characterized by the lack of laminectomy [8]. Animals were deeply anesthetized with a mixture 1:1 of Rompun (Bayer SpA, Milan, Italy; 20 mg/mL; 0.5 mL/kg) and Zoletil (Virbac Srl, Milan Italy; 100 mg/mL; 0.5 mL/kg), the back hairs were shaved, skin was disinfected with betadine, and spinal cords were exposed. Animals were mounted on a stereotaxic apparatus with spinal adaptors connected to a cortical PinPoint precision impactor device (Hatteras Instruments Inc, Cary, NC, USA) and maintained at 37 °C throughout surgery. The severe spinal trauma was induced on the basis of the following parameters: middle, round, and flat tip (#4); velocity, 3 m/s; depth, 5 mm; and dwell time, 800 ms. No laminectomy was performed. The spinal cord was injured at thoracic level 10 (T10), inducing bone fracture, as shown in Figure 1A and previously reported in References [8,17,38], and a complete absence of motor recovery, unlike from previous models [18]. Spinal vertebrae were analyzed by means of a microCT, (details in Section 4.9) [49].

The analysis of the graphical impact parameters, operated by the PinPoint software, was used to identify potential outliers. Behavioral analyses were also used to corroborate differences in injury severity within groups (as better described in behavioral tests). Slight lesions were excluded from the study based on these criteria.

Because, from our previous research [8] and preliminary results, no differences were found between sham and naïve (CTRL) animals, to minimize the animals suffering, we decided to perform all experiments by using CTRL mice, in accordance with ethical issues and Italian National law on the use of animals for research.

As postoperative care, the bladder was compressed by manual abdominal pressure twice per day until bladder function was restored; in the first 24 h, animals were maintained at 37 °C with warmed pad, rehydrated with 1 mL of ringer lactate, and, to support nutrition, humid food was inserted in the cage.

#### 4.2.2. Moderate Trauma

Anesthetized mice were mounted on a stereotaxic apparatus with spinal adaptors connected to a cortical PinPoint precision impactor device (Hatteras Instruments Inc, Cary, NC, USA) and maintained at 37 °C throughout surgery. They underwent a complete single-level laminectomy at the T10. The vertebral column was clamped and stabilized at the upper thoracic and lumbar levels, and a controlled contusion on the basis of the following parameters: middle, round, and flat tip (#4); velocity, 1 m/s; depth, 5 mm; and dwell time, 75 ms. Animals reporting a BMS score between 4 and 6 after SCI were considered moderately contused and included in the analysis.

### 4.3. Drugs

A single dose (15 pg/5 μL) of BoNT/A (150 KDa purified protein without accessory proteins) kindly gifted from Professor Cesare Montecucco (Department of Biomedical Sciences, University of Padova, Italy) or saline (5 μL) was spinally administered between the lumbar vertebrae (L4–L5) and not in the injury site (T9–T11), to avoid systemic toxin diffusion due to hemorrhagic events following the contusion. However, being BoNT/A able to be retrogradely transported [11], we can detect the proteolytic action at the injury site (as better described in the Results section). BoNT/A was injected within 1 h from surgery, using a 10 μL syringe (Hamilton #701; Biosigma Cona (Ve), Italy) equipped with a glass needle (30 μm internal diameter; Eppendorf). Injections were made at a perfusion speed of 2 μL/min, controlled by an automatic injector (KDS 310 Plus; KD Scientific; Holliston, MA, USA), and the tip of the needle was maintained inside the cord 3 min after each injection, to avoid liquid reflux. The toxin was frozen in liquid nitrogen and stored at −80 °C, in 10 mM NaHepes, 150 mM NaCl, pH 7.2. Stock solutions of BoNT/A were tested for activity in the ex vivo mouse hemidiaphragm model and in the in vitro cleavage of SNAP-25 [42]. The dose of BoNT/A was chosen on the basis of neurotoxicity studies in CNS (LD50: 0.5–1.0 × 10^−6^ mg/kg) [50] and previous behavioral studies [13,51,52,53].

### 4.4. Behavioral Test: Basso Mouse Scale (BMS)

The hindlimb locomotor function was assessed in an open field for all injured groups. Two evaluators, blind as for injury groups, scored the mice. The BMS [20] score ranges from 0 to 9, with 0 indicating complete paralysis and 9 indicating normal movement of the hindlimbs. Performance of the left and right hindlimbs was averaged in order to obtain the BMS score. Mice were tested for hindlimb functional deficits at 1, 2, 3, 4, 7, 10, 14, 17, 20, and 30 days after SCI. Only mice completely paralyzed (BMS scores = 0) after surgery were assigned to the experimental group classified as “severe” and used for the following analysis.

### 4.5. Nociceptive Tests

#### 4.5.1. Tail Flick Test

Mice were tested under the same conditions, i.e., 7:00–9:00 a.m., after 15 min of acclimating. The testing environment was cleaned thoroughly between each animal, to eliminate any odor-related stress cues. A radiant heat source with a locator light (Ugo Basile Srl, Comerio, Italy) was positioned on the tail, and the latency to withdrawal was determined. A cutoff time of 10 s was used to prevent tissue damage. The latency to a flick or flinch of the tail from the heat source was recorded with a built-in timer, which displayed reaction time in 0.01 s increments. Tests were performed three times, with a 5 min interval between each measurement, and the mean was calculated.

#### 4.5.2. Mechanical Allodynia

Mechanical allodynia induced by moderate SCI was tested, using a Dynamic Plantar Aesthesiometer (Model 37,400, Ugo Basile Srl, Comerio, Italy) described elsewhere [53]. For habituation, mice were placed 30′ before test in the experimental room and in testing plastic cages with a wire net floor 5 min before the experiment. Mechanical threshold was measured up until allodynia restoration was observable, starting from day 3. Each testing day, the withdrawal threshold of hind paws was taken as the mean of 3 consecutive measurements per paw.

### 4.6. Evaluation of Muscle Functionality

#### 4.6.1. Histological Analysis of Skeletal Muscle

For the histological analysis, 8 µm muscle cryosections were analyzed. Cryosections were fixed and permeabilized with 100% acetone for 1 min at RT. Muscle sections were then blocked for 1 h with a solution containing 4% BSA in PBS. The anti-Laminin (#L9393, Sigma-Aldrich Merck, Darmstadt, Germany, 1:400) and anti-Neurofilament-L (#2835S, Cell Signaling, Leiden, the Netherlands 1:200) immunostaining was performed overnight at 4 °C, and then the antibody binding specificity was revealed by using secondary antibodies coupled to Alexa Fluor 647 or Alexa Fluor 488 respectively. Neuromuscular junction was stained by using α-Bungarotoxin Alexa FluorTM 594 conjugate (#B13423, Invitrogen, Thermo Fisher Scientific Waltham, MA USA 1:500). Sections were incubated with DAPI in PBS for 5 min for nuclear staining, washed in PBS, and mounted with glycerol 3:1 in PBS. Alternative muscle section was stained, using Hematoxylin–Eosin standard protocol [54]. The transverse sections were visualized on a Zeiss confocal microscope, and myofibers cross-sectional (CSA) was quantified using ImageJ^®^ software (National Institutes of Health, Bethesda, MD, USA). The entire muscle section was analyzed. Values are shown as mean CSA or percentage of frequency distribution. For nicotinamide adenosine dehydrogenase–tetrazolium reductase (NADH–TR) staining, muscle cryosections were incubated for 15 min with a solution containing NADH and nitrotetrazolium blue (# N8129 and #N6876, Sigma-Aldrich Merck, Darmstadt, Germany) in Tris-HCl (pH 7.4), at 37 °C, according to the standard protocol [55].

#### 4.6.2. RT-PCR and qPCR

Total RNA from skeletal muscle tissue was extracted with TRIzol and retro-transcribed with a Taqman reverse transcription kit (#N8080234, Applied Biosystems Foster City, CA, USA), following the manufacturer’s indication. Then, qRT-PCR was performed by using SYBR Green Master mix (#4309155, Applied Biosystems, Foster City, CA, USA), following manufacturer indications. Relative expression values were normalized to the housekeeping gene Gapdh. The primer used was: Gapdh FW CACCATCTTCCAGGAGCGAG, Gapdh RV CCTTCTCCATGGTGGTGAAGAC, Atrogin-1 FW TGAGCGACCTCAGCAGTTAC, Atrogin-1 RV TTCTCTTCTTGGCTGCGACG, MuRF1 FW AACTTGTGGAGACCGCCATC, MuRF1 RV TGGAGGCTTCTACAATGCTCTT, Myogenin FW GTCCCAACCCAGGAGATCATTT, and Myogenin RV CAGACATATCCTCCACCGTGA.

### 4.7. Immunohistochemistry

Seven, 30, or 60 days post-SCI, three mice from each experimental group were sacrificed for immunohistochemistry analysis and perfused with saline, followed by 4% paraformaldehyde in phosphate buffer saline (PBS, pH 7.4). Brain (only at D30), thoracic (T1–T13), and lumbar (L2–L5) spinal cord of mice were harvested and kept in immersion for 48 h in 4% paraformaldehyde in phosphate buffer saline (PBS, pH 7.4), after cryoprotection with solution of 30% (*w*/*v*) sucrose in PBS, and maintained at −80 °C. Cryostat sections of 40 μm were taken. For double IF staining, different sections were incubated for 48 h, at room temperature, with primary antibodies (see Table S1) in Triton 0.3%. Sections were then washed in PBS and incubated for 2 h, at room temperature, with secondary antibodies (see Table S1). Sections were again washed in PBS and incubated for 10 min with Bisbenzimide (Hoechst 33258, 1:1000, Jackson ImmunoResearch, Cambridge House, Ely, UK) to stain nuclei. After washing in PBS, sections were mounted on glass slides.

#### 4.7.1. Histological Analysis of Spinal Cord Tissue

Low- (10×; 20× objective) and high-magnification (63× objective) IF images from immunostained sagittal spinal cord sections were captured with laser scanning confocal microscopy, using a TCS SP5 microscope (Leica Microsystems, Milan, Italy) connected to digital-camera diagnostic instruments operated by LAS AF lite software of Leica Microsystems (free download www.leica-mycrosystems.com). All analyses were performed in sequential scanning mode, to rule out cross-bleeding between channels. Quantification was performed by using the ImageJ software (version 1.41; National Institutes of Health, Bethesda, MD, USA). The number of IF-positive cells (nuclei) was automatically counted with mark and count tool, and then the mean for each group of mice was calculated. Fluorescence was quantified with the RGB (red, green, and blue) method, which converts RGB pixels to brightness value or by Integrated Density of fluorescence of the selected area and colocalization area by using the Manders’ coefficient [56]. This analysis uses an iterative procedure to determine what pair of thresholds for the 2 channels of the scatterplot gives a Manders’ correlation coefficient (r) of zero for the pixels below the thresholds (Evans, 34: 0–0.19 “very weak”, 0.20–0.39 “weak”, 0.40–0.59 “moderate”, 0.60–0.79 “strong”, and 0.80–1.0 “very strong”). A double fluorescence image (red and green dyes) was split in RGB channels and analyzed. For each colocalization analysis, scatterplots were generated by the Image J program: The intensity of channel 1 pixels is used as the *x*-coordinate, whereas the intensity of the channel 2 pixels as the *y*-coordinate.

Three animals per group were considered, and two/three sections per animal were randomly selected and analyzed. For all groups, sections of ventral horns (VH), distal from the impact zone (3–5 mm rostro-caudal to lesion epicenter) and at the injury site (peri-lesioned area and epicenter T9–T11) were separately analyzed. Naïve animals represent the CTRL group.

#### 4.7.2. Lesion Size (Microcystic Degeneration and Neuronal Survival)

Saline and BoNT/A spinal sections (10×) stained for DAPI were digitally converted (ImageJ software National Institutes of Health, Bethesda, MD, USA) in binary images (black-and-white). Black areas (microcysts) were marked with ward (tracing) tool and automatically measured (pixels). Perimeter of the spinal slice was marked with freehand selection tool and the correspondent area (spinal section) automatically measured (pixels). The overall damaged area (cavity) was calculated as percentage of the spinal section area as following: cavity dimension/spinal section*100. For each animal, at least two slices were analyzed. Neuronal survival was assessed by counting the NeuN-positive cells in the VH caudal to the lesion (L2–L5).

#### 4.7.3. Morphometric Analysis

Morphometric analysis was performed on high-resolution images, acquisition criteria: 63× objective, zoom 3 or 2×, 1024 × 1024 frame, 10 Hz. Each image was transformed in binary modality, in order to obtain a digital image where the outline of cell silhouettes was identified and automatically measured for astrocytes while microglia was singularly counted and divided about the different morphology (R: resting (ramified), H/B: hyperactive/bushy, and U/A: un-ramified/ameboid).

### 4.8. Western Blot

For the analysis of the myelin basic protein (MBP) expression, a total 10 µg of spinal cord samples (T9–T11, Impact zone), from 3 experimental groups (naïve, saline and BoNT/A), at two different time points (7 and 30 days), were homogenized and centrifuged at 4 °C for 20 min at 13,000 rpm, and then the supernatants were stored at −20 °C. Proteins were applied to sodium dodecyl sulfate–polyacrylamide gel electrophoresis and electroblotted on a nitrocellulose membrane. Membranes were incubated overnight at 4 °C, with the following primary antibodies: mouse monoclonal to Myelin Basic Protein (MBP101, Abcam, Cambridge, UK) (1:500) and mouse monoclonal to β actin (1:1000) (Abgent, San Diego, CA, USA). Horseradish peroxidase-conjugated anti-mouse IgG (Jackson Immuno Research Cambridge House, Ely, UK) (1:40000) was used as secondary antibody. The βactin bands intensity was used as a control for equal protein loading and measured for densitometric analysis, using ImageJ 1.49r software (Wayne Rasband, National Institutes of Health, Bethesda, MA, USA).

### 4.9. Micro Xray Computed Tomography (MicroCT)

Spine sections of the 3 experimental groups (naïve, saline, and BoNT/A), containing the damaged area, were soaked in a potassium iodine contrast agent, 0.1N (*v*/*v*) Lugol solution (Sigma), at room temperature, in the dark, for 3 months, replacing the solution several times during the exposure. The acquisition of tomographic datasets was performed through a high-resolution 3D Micro-CT Imaging System (Skyscan 1172G Bruker, Kontich, Belgium), at 12 µm pixel/size (2 × 2) and the reconstructions, using built-in NRecon Skyscan Software (Version 1.6.6.0, Bruker).

Tridimensional volumes were analyzed by using 3D Visualization Software CTvox v. 2.5 (Bruker) for creating images and movies in grayscale and reversed B/W. Size measurements were calculated as the average of 5 manual evaluations, performed using Bruker CT-Analyser 1.13 Software, at the level of the same transverse slide for each sample, selected from the samples with the bone fracture. The entire transverse dataset was aligned based on the orientation of the fractured vertebra, and therefore the same vertebra for each specimen, using the Atlas of the Mouse Brain and Spinal Cord (Sidman–Angevine–Pierce, 1971) for guidance to accurately identify the correct orientation of the virtual sections.

### 4.10. Glycemia and Triglycerides Measurement

Blood glucose and triglycerides were measured by using a Multicare Test Strips apparatus (Biochemical Systems International, Arezzo, Italy), by tail clipping in baseline (BL) conditions and at different time points post-SCI (2 h, 4 h, 24 h, 48 h, day 3, day 4, and day 7) both in saline and BoNT/A-treated mice.

### 4.11. Experimental Design and Statistical Analyses

Experimental groups belong to the same statistical population with similar variance. The sample size, relatively to behavioral experiments, was preventively calculated with G*Power (Software, Heinrich-Heine-Universität Düsseldorf, Germany) (for ANOVA repeated measures, 3 groups, including control, and 10 measurements), and the estimation for a medium effect (0.25) is *N* = 27 (about 9 for group). With the regard of immune- and biochemical experiments, sample size was estimated according to previous experience, using the models described.

Experimental data are expressed as mean ± SEM. Depending on data, group comparisons were carried out by means of analyses of variance (ANOVAs one-way or two-way for repeated measures), by Student’s *t*-test, or by non-parametric analysis Kruskal–Wallis for small samples (*N* < 5 animals) and groups >3. Post hoc comparisons were made with the Tukey–Kramer or Dunn test. Considering the highly complex and heterogeneous statistical analyses utilized, we reported the statistical test used for each experiment in the Results section. Data were considered statistically significant at *p* < 0.05. Staview SAS version 5.0, Cary NC, USA, R (R Foundation, Wirtschaftsuniversität Wien, Austria), and GraphPad Prism (San Diego, CA, USA) were used for data analysis.

## Figures and Tables

**Table 1:** Main SCI-induced degenerative events during acute, intermediate, and chronic phases.

Acute Phase (Initial Physical Insult)	Intermediate Phase (Cascade of Detrimental Events)	Chronic Phase (Irreversible Damage)
Spinal shock and hemorrhage	Microcystic formation	Mature cystic formation
Ischemia and cell death	Apoptosis	Extending apoptosis out of injury site
Edema, thrombosis and inflammation	Edema, immune cells invasion, microglia response, cytokines release	Demyelination
Glutamatergic excitotoxicity	Astrocytes hypertrophy and glia scar formation	Scarring
	Demyelination	
Electrolytes and metabolic alterations	Electrolytes and metabolic alterations	Regeneration/sprouting

## Supplementary Materials

The following are available online at https://www.mdpi.com/2072-6651/12/8/491/s1. Figure S1: Expression of Caspase 3 and OLIG1, in peri-lesioned area seven days after SCI, Table S1: Antibodies list, Video S1: Severe SCI induces permanent hindlimbs paralysis. Video shows a saline-treated mouse 3, 5, 7, 14, 21 and 30 days after spinal trauma,Video S2: Effects of BoNT/A treatment on motor functional recovery after severe SCI. Video shows a BoNT/A-treated mouse 3, 5, 7, 14, 21 and 30 days after spinal trauma, Video S3: 3D video-reconstruction (MicroCT) of a naïve mouse spinal cord, Video S4: 3D video-reconstruction (MicroCT) of a saline-treated mouse spinal cord 30 days after trauma, Video S5: 3D video-reconstruction (MicroCT) of a BoNT/A-treated mouse spinal cord 30 days after trauma.

## References

[1] Consortium for Spinal Cord Medicine (2008). Early acute management in adults with spinal cord injury. J. Spinal Cord Med..

[2] Kumar R., Lim J., Mekary R.A., Rattani A., Dewan M.C., Sharif S.Y., Osorio-Fonseca E., Park K.B. (2018). Traumatic spinal injury: Global epidemiology and worldwide volume. World Neurosurg..

[3] Lee B.B., Cripps R.A., Fitzharris M., Wing P.C. (2013). The global map for traumatic spinal cord injury epidemiology: Update 2011, global incidence rate. Spinal Cord.

[4] Kim Y.H., Ha K.Y., Kim S.-I. (2017). Spinal cord injury and related clinical trials. CIOS Clin. Orthop. Surg..

[5] Alizadeh A., Dyck S.M., Karimi-Abdolrezaee S. (2015). Myelin damage and repair in pathologic CNS: Challenges and prospects. Front. Mol. Neurosci..

[6] Hutson T.H., Di Giovanni S. (2019). The translational landscape in spinal cord injury: Focus on neuroplasticity and regeneration. Nat. Rev. Neurol..

[7] Boccella S., Vacca V., Errico F., Marinelli S., Squillace M., Guida F., Di Maio A., Vitucci D., Palazzo E., De Novellis V. (2015). D-aspartate modulates nociceptive-specific neuron activity and pain threshold in inflammatory and neuropathic pain condition in mice. BioMed Res. Int..

[8] Marinelli S., Vacca V., De Angelis F., Pieroni L., Orsini T., Parisi C., Soligo M., Protto V., Manni L., Guerrieri R. (2019). Innovative mouse model mimicking human-like features of spinal cord injury: Efficacy of Docosahexaenoic acid on acute and chronic phases. Sci. Rep..

[9] Fonfria E., Maignel J., Lezmi S., Martin V., Splevins A., Shubber S., Kalinichev M., Foster K., Picaut P., Krupp J. (2018). The expanding therapeutic utility of botulinum neurotoxins. Toxins.

[10] Antonucci F., Rossi C., Gianfranceschi L., Rossetto O., Caleo M. (2008). Long-distance retrograde effects of botulinum neurotoxin A. J. Neurosci..

[11] Marinelli S., Vacca V., Ricordy R., Uggenti C., Tata A.M., Luvisetto S., Pavone F. (2012). The analgesic effect on neuropathic pain of retrogradely transported botulinum neurotoxin A involves Schwann cells and Astrocytes. PLoS ONE.

[12] Caleo M., Restani L. (2018). Direct central nervous system effects of botulinum neurotoxin. Toxicon.

[13] Marinelli S., Luvisetto S., Cobianchi S., Makuch W., Obara I., Mezzaroma E., Caruso M., Straface E., Przewlocka B., Pavone F. (2010). Botulinum neurotoxin type A counteracts neuropathic pain and facilitates functional recovery after peripheral nerve injury in animal models. Neuroscience.

[14] Pavone F., Luvisetto S. (2010). Botulinum neurotoxin for pain management: Insights from animal models. Toxins.

[15] Cobianchi S., Jaramillo J., Luvisetto S., Pavone F., Navarro X. (2017). Botulinum neurotoxin A promotes functional recovery after peripheral nerve injury by increasing regeneration of myelinated fibers. Neuroscience.

[16] Pirazzini M., Rossetto O., Eleopra R., Montecucco C. (2017). Botulinum neurotoxins: Biology, pharmacology, and toxicology. Pharmacol. Rev..

[17] Marinelli S., Pavone F., Luvisetto S., Vacca V. (2015). A New Therapeutic Use of the Botulinum Neurotoxin Serotype A. International Patent.

[18] Kundi S., Bicknell R., Ahmed Z. (2013). Spinal cord injury: Current mammalian models. Am. J. Neurosci..

[19] Cheriyan J., Ryan D.J., Weinreb J.H., Paul J.C., Lafage V., Kirsch T., Errico T.J. (2014). Spinal cord injury models: A review. Spinal Cord.

[20] Basso D.M., Fisher L.C., Anderson A.J., Jakeman L., McTigue D.M., Popovich P.G. (2006). Basso mouse scale for locomotion detects differences in recovery after spinal cord injury in five common mouse strains. J. Neurotrauma.

[21] Yang T., Dai Y., Chen G., Cui S. (2020). Dissecting the dual role of the glial scar and scar-forming astrocytes in spinal cord injury. Front. Cell Neurosci..

[22] Yamamotová A., Sramkova T., Rokyta R. (2009). Intensity of pain and biochemical changes in blood plasma in spinal cord trauma. Spinal Cord.

[23] Kobayakawa K., Kumamaru H., Saiwai H., Kubota K., Ohkawa Y., Kishimoto J., Yokota K., Ideta R., Shiba K., Tozaki-Saitoh H. (2014). Acute hyperglycemia impairs functional improvement after spinal cord injury in mice and humans. Sci. Transl. Med..

[24] Laclaustra M., van den Berg E.L.M., Hurtado-Roca Y., Castellote J.M. (2015). Serum lipid profile in subjects with traumatic spinal cord injury. PLoS ONE.

[25] Mills C.D., Fullwood S.D., Hulsebosch C.E. (2001). Changes in Metabotropic Glutamate receptor expression following spinal cord injury. Exp. Neurol..

[26] Li N., Leung G.K.K. (2015). Oligodendrocyte precursor cells in spinal cord injury: A review and update. BioMed Res. Int..

[27] Bartholdi D., Schwab M.E. (1998). Oligodendroglial reaction following spinal cord injury in rat: Transient upregulation of MBP mRNA. Glia.

[28] Marichal N., Reali C., Trujillo-Cenóz O., Russo R.E. (2017). Spinal Cord Stem Cells in Their Microenvironment: The Ependyma as a Stem Cell Niche. Retinal Degenerative Diseases.

[29] Sandri M. (2013). Protein breakdown in muscle wasting: Role of autophagy-lysosome and ubiquitin-proteasome. Int. J. Biochem. Cell Boil..

[30] Moresi V., Williams A.H., Meadows E., Flynn J.M., Potthoff M.J., McAnally J., Shelton J.M., Backs J., Klein W.H., Richardson J.A. (2010). Myogenin and class II HDACs control neurogenic muscle atrophy by inducing E3 Ubiquitin Ligases. Cell.

[31] Rhéaume C., Cai B.B., Wang J., Fernandez-Salas E., Aoki K.R., Francis J., Broide R.S. (2015). A highly specific Monoclonal antibody for botulinum neurotoxin Type A-Cleaved SNAP25. Toxins.

[32] Vogelaar C.F. (2016). Extrinsic and intrinsic mechanisms of axon regeneration: The need for spinal cord injury treatment strategies to address both. Neural Regen. Res..

[33] Yiu G., He Z. (2006). Glial inhibition of CNS axon regeneration. Nat. Rev. Neurosci..

[34] Karimi-Abdolrezaee S., Billakanti R. (2012). Reactive Astrogliosis after spinal cord injury—Beneficial and detrimental effects. Mol. Neurobiol..

[35] Hausmann O.N. (2003). Post-traumatic inflammation following spinal cord injury. Spinal Cord.

[36] Ju G., Wang J., Wang Y., Zhao X. (2014). Spinal cord contusion. Neural Regen. Res..

[37] Walters E.T. (2014). Neuroinflammatory contributions to pain after SCI: Roles for central glial mechanisms and nociceptor-mediated host defense. Exp. Neurol..

[38] Shi J., Dong B., Mao Y., Guan W., Cao J., Zhu R., Wang S. (2016). Review: Traumatic brain injury and hyperglycemia, a potentially modifiable risk factor. Oncotarget.

[39] Ming G.-L., Song H. (2011). Adult neurogenesis in the mammalian brain: Significant answers and significant questions. Neuron.

[40] Ke Y., Chi L., Xu R., Luo C., Gozal D., Liu R. (2005). Early response of endogenous adult neural progenitor cells to acute spinal cord injury in mice. Stem Cells.

[41] Xu R., Wu C., Tao Y., Yi J., Yang Y., Zhang X., Liu R. (2008). Nestin-positive cells in the spinal cord: A potential source of neural stem cells. Int. J. Dev. Neurosci..

[42] Qin Y., Zhang W., Yang P. (2014). Current states of endogenous stem cells in adult spinal cord. J. Neurosci. Res..

[43] Martino G., Pluchino S. (2006). The therapeutic potential of neural stem cells. Nat. Rev. Neurosci..

[44] Dooley D., Vidal P., Hendrix S. (2014). Immunopharmacological intervention for successful neural stem cell therapy: New perspectives in CNS neurogenesis and repair. Pharmacol. Ther..

[45] Finnerup N.B. (2017). Neuropathic pain and spasticity: Iintricate consequences of spinal cord injury. Spinal Cord.

[46] Giangregorio L., McCartney N. (2006). Bone loss and muscle atrophy in spinal cord injury: Epidemiology, fracture prediction, and rehabilitation strategies. J. Spinal Cord Med..

[47] Mignone J.L., Kukekov V., Chiang A.-S., Steindler D., Yenikolopov G. (2004). Neural stem and progenitor cells in nestin-GFP transgenic mice. J. Comp. Neurol..

[48] Zimmermann M. (1983). Ethical guidelines for investigations of experimental pain in conscious animals. Pain.

[49] Ermakova O.V., Orsini T., Gambadoro A., Chiani F., Tocchini-Valentini G.P. (2017). Three-dimensional microCT imaging of murine embryonic development from immediate post-implantation to organogenesis: Application for phenotyping analysis of early embryonic lethality in mutant animals. Mamm. Genome.

[50] Schiavo G., Montecucco C. (1995). Tetanus and botulism neurotoxins: Isolation and assay. Methods Enzymol..

[51] Luvisetto S., Rossetto O., Montecucco C., Pavone F. (2003). Toxicity of botulinum neurotoxins in central nervous system of mice. Toxicon.

[52] Luvisetto S., Marinelli S., Lucchetti F., Marchi F., Cobianchi S., Rossetto O., Montecucco C., Pavone F. (2006). Botulinum neurotoxins and formalin-induced pain: Central vs. peripheral effects in mice. Brain Res..

[53] Luvisetto S., Marinelli S., Cobianchi S., Pavone F. (2007). Anti-allodynic efficacy of botulinum neurotoxin A in a model of neuropathic pain. Neuroscience.

[54] Fiacco E., Castagnetti F., Bianconi V., Madaro L., De Bardi M., Nazio F., D’Amico A., Bertini E., Cecconi F., Puri P.L. (2016). Autophagy regulates satellite cell ability to regenerate normal and dystrophic muscles. Cell Death Differ..

[55] Madaro L., Marrocco V., Carnio S., Sandri M., Bouchè M. (2013). Intracellular signaling in ER stress-induced autophagy in skeletal muscle cells. FASEB J..

[56] Manders E.M., Stap J., Brakenhoff G.J., Van Driel R., Aten J.A. (1992). Dynamics of three-dimensional replication patterns during the S-phase, analysed by double labelling of DNA and confocal microscopy. J. Cell Sci..

